# A High-Performance Film for Detecting Malachite Green

**DOI:** 10.3390/mi17030365

**Published:** 2026-03-18

**Authors:** Jiao Yang, Liqin Cui, Yibo Zhao, Xiaoping Wu

**Affiliations:** 1 College of Microelectronics and Artificial Intelligence, Kaili University, Kaili 556011, China; 2 College of Science, Kaili University, Kaili 556011, China; 3 School of Optoelectronic Engineering, Xi’an Technological University, Xi’an 710021, China

**Keywords:** film, malachite green, nano-network structure, aquatic products

## Abstract

The residual malachite green (MG) in aquatic products poses a severe threat to human health, thus an urgent need exists for the establishment of a rapid and accurate analytical method. In this work, a high-performance film based on a nano-network structure was developed for the highly sensitive detection of MG. This film employed the nanonetwork structure as its sensing substrate, and the network structure with a high specific surface area enabled efficient enrichment of MG molecules. The silver nanoparticles uniformly modified on the surface could produce a remarkable localized surface plasmon resonance effect, thereby significantly enhancing the signals of MG molecules adsorbed on its surface. The results showed that the film exhibited a low limit of detection (LOD) of 8.8 pM for MG, with a linear range from 10 to 5000 pM. In the detection of aquatic products, this film successfully achieved the rapid and accurate determination of MG in aquatic products, showing an excellent potential for practical applications. The nanonetwork-structured film developed in this work provides a reliable and sensitive technical solution for the trace detection of MG in aquatic products.

## 1. Introduction

Malachite green (MG) is a dye widely used in the printing and dyeing industry. Owing to its low cost and strong bactericidal activity, it has also been extensively applied in aquaculture farms [1]. Unfortunately, human consumption of fish contaminated with MG increases the risk of carcinogenesis and can induce fetal malformation in pregnant women [2]. Due to its severe side effects, the United States, the European Union and China have now prohibited the use of MG in aquaculture [3]. Nevertheless, illegal use of MG by some fishermen still persists because of its easy access and low cost. Therefore, a rapid and sensitive method is required for the detection of MG in aquatic products.

In recent years, researchers have employed immunoassay, high-performance liquid chromatography (HPLC) [4], gas chromatography (GC) [5], electrochemical methods [6] and surface-enhanced Raman scattering (SERS) [7] for MG detection. Among these methods, electrochemical detection has been widely adopted by most researchers due to its advantages of simple operation and rapid response. Imtiaz et al. fabricated a COOH-functionalized multi-walled carbon nanotube (COOH-fMWCNT) and TiO_2_/ZnO nanocomposite sensor for the detection and photocatalytic degradation of MG. This sensor exhibited high sensitivity with a limit of detection (LOD) of 0.34 × 10^−9^ [8]. Wang et al. prepared a high nitrogen-doped carbon nanosheet sensor, which successfully detected MG in tap water with a minimum LOD of 1.32 × 10^−8^ M [9]. Li et al. synthesized a 2D conductive M_3_(HHTP)_2_ nanorod sensor, which achieved MG detection in fish meat with an LOD of 1.34 × 10^−9^ M [10]. However, due to the low electrochemical activity of MG, further improvement of the electrochemical detection limit is still needed for this banned aquaculture drug.

Compared with electrochemical detection, SERS exhibits superior detection performance, which enables rapid and selective detection at the single-molecule level. SERS sensors utilize the localized surface plasmon resonance (LSPR) effect of metal nanoparticles such as Au, Ag and Cu to significantly enhance the Raman spectral signals of MG through electromagnetic field enhancement, thus realizing the detection of low-concentration MG. In recent years, many researchers have reported the fabrication of SERS sensors for MG detection. Zhou et al. prepared an Au@Ag nanorod sensor for MG detection in crayfish with an LOD of 1.58 × 10^−9^ M [11]. Zhao et al. fabricated a MIL-101-MA@Ag sensor for MG detection with an LOD of 9.5 × 10^−11^ M [12]. Gao et al. designed a droplet-based dual-mode sensor for MG detection on the basis of silver nanoparticles (AgNPs). Under optimized conditions, this sensor achieved an LOD of 2.26 × 10^−9^ M for MG detection via the SERS method [13]. Liu et al. prepared a SERS sensor with a heterostructured nano-pineapple (NPP) morphology, which exhibited an LOD of 7.8 × 10^−11^ M for MG detection [14]. However, the aforementioned sensors have the disadvantages of complex preparation processes and weak detection performance. Therefore, developing a sensor with a simple fabrication process and robust detection capabilities holds significant research and practical value for the rapid detection of MG.

**Figure 1:**
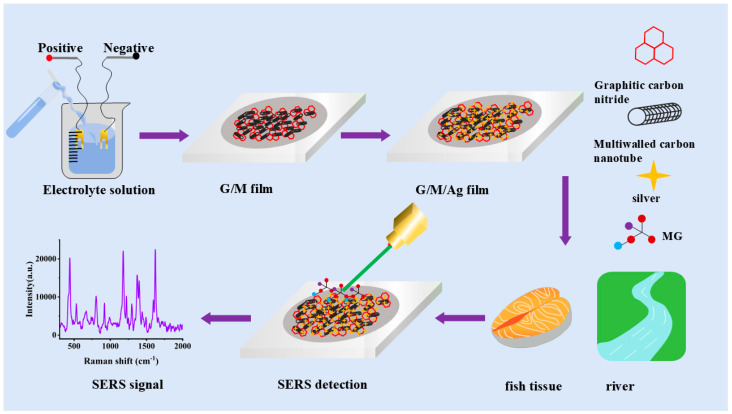
The process of preparing the G/M/Ag sensor for detecting MG.

As shown in Figure 1, a high-performance SERS sensor with a nano-network structure was designed and constructed in this work for the ultra-sensitive and selective detection of MG residues in aquatic products. The sensor adopted a graphite-phase carbon nitride/multi-walled carbon nanotube (G/M) composite structure as the sensor framework, and Ag nanoflowers with a tip-enhancement effect were uniformly deposited on its surface via electroreduction, finally forming a G/M/Ag ternary composite sensing interface with hierarchically porous characteristics. This G/M composite sensor has an extremely high specific surface area, which provides abundant adsorption sites for target molecules. In addition, it endows the sensor with excellent electron transport capability by virtue of the 3D interconnected conductive network constructed by MWCNTs. This characteristic enables it to efficiently capture and enrich MG molecules, and simultaneously significantly improve the chemical enhancement (CM) performance of the sensor by promoting the charge transfer (CT) process at the interface. In addition, the Ag nanoflowers loaded on the surface of the G/M network generate a strong localized surface plasmon resonance (LSPR) effect, forming a large number of electromagnetic (EM) hot spots at the tips and gaps of the nanoflowers, thus producing an extremely strong electromagnetic field enhancement effect on MG molecules adsorbed in their vicinity. The synergistic effect of the CM and EM mechanisms endows the sensor with an excellent amplification effect on the Raman signals of MG. This sensor is used to detect MG residues in aquatic products, providing a reliable technical platform for on-site monitoring of aquatic product safety.

## 2. Materials and Methods

### 2.1. Materials

Titanium sheets (99.9%) were purchased from Chenghe County Tengfeng Metal Materials Co., Ltd. (Handan, China). Ethanol (99.7%), silver nitrate (99.8%), citric acid (98.0%), graphitic carbon nitride (95.0%), multiwalled carbon nanotubes (95%, ID: 3–5 nm, OD: 8–15 nm, Length: 50 μm), sodium sulfate (99.0%) and sodium fluoride (98.0%) were obtained from Sinopharm Chemical Reagent Co., Ltd. (Shanghai, China).

### 2.2. Experimental Techniques

To characterize the physical and chemical properties of the films, the surface micromorphology was observed via a scanning electron microscope (SEM, SU1510, Hitachi, Tokyo, Japan). The accelerating voltage used in the SEM test was 10.0 kV. Raman spectra were collected using a fully automatic Raman spectrometer (XploRA PLUS, HORIBA Jobin Yvon, Paris, France) with a 532 nm wavelength laser as the excitation source. The laser power of 15 mW was focused on the sample using an objective lens with 50x magnification and 0.5 numerical aperture. The diffraction grating was 600 L/mm. The accumulation time was 3 s with 3 repetitions.

### 2.3. Preparation of the G/M/Ag Sensor

The processes of electrodeposition and anodization were carried out simultaneously in an electrolyte containing 1 g/L graphitic carbon nitride, 0.5 g/L multi-walled carbon nanotubes, 1 mol/L Na_2_SO_4_ and 0.5 wt% NaF. A titanium sheet was used as the anode and a platinum (Pt) sheet as the cathode. The G/M film was obtained via deposition at a constant voltage of 10 V for 40 min [15]. Subsequently, the as-prepared G/M film was immersed in a mixed solution consisting of 1 mM AgNO_3_ and 0.1 M citric acid. With the G/M film as the anode and Pt as the cathode, electrodeposition was conducted at −0.8 V for 30 min to fabricate the G/M/Ag sensor [16].

### 2.4. Preparation of Real Samples

The preparation method for fish tissue samples was referred to the reported protocol [17]. After pretreatment of the real fish samples, MG was spiked into the samples to prepare spiked solutions with different MG concentrations (500, 1000, 2000, 3000, 5000 pM). Due to the presence of a large amount of complex environmental matrices in the river water, we chose the river water as the representative sample. In this work, river water samples were collected from the Bala River, and MG was added to the river water to prepare spiked river water samples with a series of MG concentrations (300, 1000, 2000, 3000, 5000 pM).

## 3. Results

### 3.1. The Morphology of the G/M/Ag Sensor

**Figure 2:**
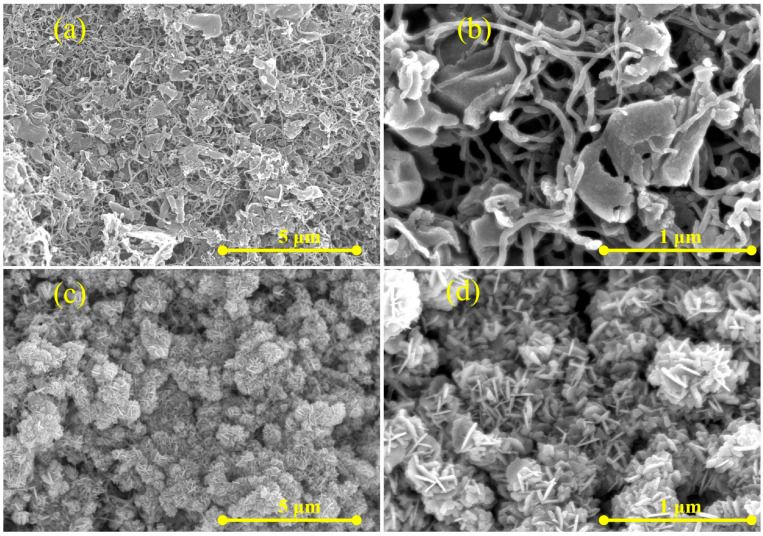
(**a**) SEM morphology of the G/M structure. (**b**) Magnified view of the SEM morphology of the G/M structure. (**c**) SEM morphology of the G/M/Ag structure. (**d**) Magnified view of the SEM morphology of the G/M/Ag structure.

The G/M/Ag ternary composite exhibited a three-dimensional nano-network structure, which served as the origin of its high-performance SERS detection capability. As shown in Figure 2a,b, multi-walled carbon nanotubes were intertwined with each other, penetrated and anchored between the g-C_3_N_4_ nanosheets, thus forming a 3D interconnected conductive network skeleton. In Figure 2c,d, silver nanoparticles (Ag NPs) were densely and uniformly loaded on the surface of g-C_3_N_4_ nanosheets, as well as on the tube walls and junctions of MWCNTs. It could be seen that the diameter of the nanostructure formed by AgNPs nanoparticles was approximately 500 nm. This composite structure created a large number of gaps and interfaces on the sensor surface, thereby generating an extremely high density of localized electromagnetic field “hot spots” under laser excitation. When MG molecules were captured in the dense “hot spot” regions formed by AgNPs, they were simultaneously subjected to an intense electromagnetic field enhancement. In addition, the G/M/Ag NPs structure could undergo charge transfer with molecules upon laser stimulation. The chemical enhancement (CM) induced by charge transfer and electromagnetic enhancement (EM) could further amplify the intensity of the Raman signals of MG.

### 3.2. Detection of MB Molecules by G/M/Ag Sensor

The morphology and size of AgNPs directly affect the detection capability of the sensor. In this study, to optimize the SERS performance of the sensor, we investigated its enhancement ability for MG by adjusting the controlling the deposition tim of AgNPs. We selected the intensity of the Raman peak at 1620 cm^−1^ of the MG molecule to investigate the enhancement properties of the sensor.

**Figure 3:**
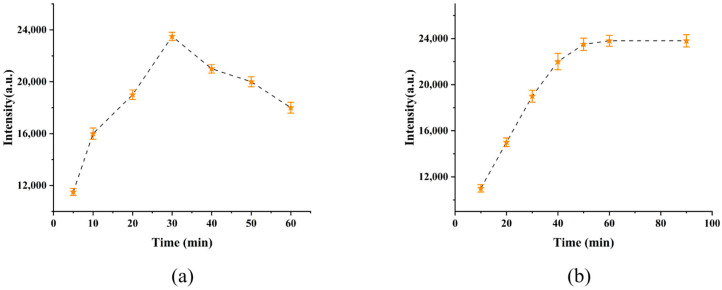
(**a**) The influence of silver deposition time on SERS intensity on the G/M surface. (**b**) The SERS intensity of the G/M/Ag sensor after being immersed in 5000 pM MG for different periods of time.

As shown in Figure 3a, with the extension of deposition time, the rough surface of AgNPs could generate strong LSPR under laser stimulation to enhance molecules, thereby improving the enhancement ability of the film for 5000 pM MG molecules. However, when the deposition time exceeded 30 min, the silver nanoparticles on the surface aggregated together, resulting in a decrease in the Raman intensity of MG at 1620 cm^−1^. Therefore, the optimal AgNPs deposition time of G/M/Ag sensor was determined to be 30 min. In addition, this work also analyzed the adsorption performance of the sensor for MG under different immersion times. Furthermore, the results in Figure 3b indicated that after the soaking time exceeded 50 min, the MG molecules on the surface of the sensor reached saturation, and the intensity of MG at 1620 cm^−1^ also tended to stabilize. This was mainly attributed to the three-dimensional nano-network structure on the sensor surface, which enabled the rapid capture of MG molecules. To test the detection capability of the G/M/Ag sensor for MG, the sensor was used to detect MG at different concentrations (10, 100, 1000, 3000, 5000, 8000, 15,000, 20,000, 30,000, 50,000 pM) and collect the corresponding SERS spectra of MG.

**Figure 4:**
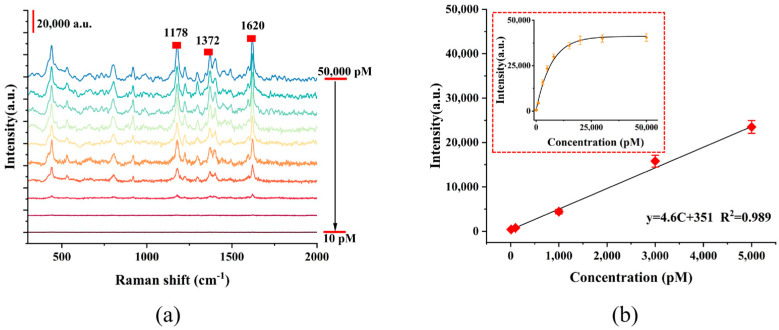
(**a**) Detection of different concentrations of MB using G/M/Ag sensor. (**b**) The relationship between the SERS intensity at 1620 cm^−1^ and the concentration of MG.

The results in Figure 4a showed that with the increase in MG concentration, the number of molecules adsorbed on the surface of the composite film exhibited an obvious accumulation trend. When irradiated by laser, intense surface plasmon coupling occurred between the nanoparticles in the film, forming highly localized electromagnetic field “hot spots”. At the same time, the interfacial electron transfer process between the film material and MG molecules was significantly promoted, and the synergistic effect of EM and CM jointly achieved a remarkable amplification of the Raman signals of the MG molecule. The 1620 cm^−1^ band was assigned to the stretching vibrations of double C=C bonds (C_ph_=C_ph_) in the benzene ring. The 1178 cm^−1^ band was attributed to the in-plane bending vibration of CCH in the benzene ring (δ(C_ph_C_ph_H_ph_)). The 1378 cm^−1^ band was identified as the stretching vibration of the single bond between the carbon atom of the benzene ring and the nitrogen atom (v(C_ph_-N)) [1]. In this work, SERS tests were conducted on MG solutions with different concentrations using the G/M/Ag sensor, and the intensity of the characteristic peak at 1620 cm^−1^ was compared to the corresponding concentration. The obtained results are shown in Figure 4b. Within the concentration range of 10 pM to 5000 pM, the signal intensity of the characteristic Raman peak and the MG concentration exhibited a good linear dependence (R^2^ > 0.9). Based on the signal-to-noise ratio (S/N = 3), the LOD of the sensor for MG was calculated to be 8.8 pM. The above results verified the quantitative detection capability of the composite film for MG over a wide concentration range, demonstrated its excellent sensitivity and detection reliability, and indicated its potential for the detection of trace MG in real samples.

**Figure 5:**
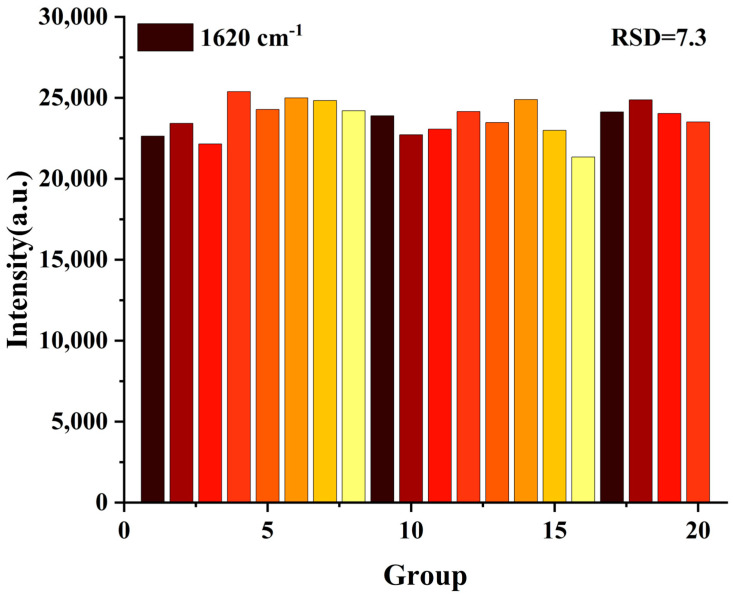
Randomly selected 20 points on the surface of the G/M/Ag sensor to collect the SERS intensity of 5000 pM MG at 1620 cm^−1^.

To evaluate the signal repeatability of the composite film in detecting MG molecules—a key index reflecting the uniformity and reliability of the sensor, 20 different positions were randomly selected from the surface of the film for testing in this work to reflect the overall performance of the film. In this study, we selected the highest concentration of 5000 pM MG within the linear range for the test. The Raman peak at 1620 cm^−1^ was selected for study due to its most stable SERS intensity among the MG molecules. Figure 5 presented the signal intensity distribution of the characteristic peak at 1620 cm^−1^ among the 20 testing points. After calculation, the relative standard deviation (RSD) of the MG signal detected by the film was 7.3%, indicating that the composite film had excellent repeatability and stable performance in practical detection.

### 3.3. G/M/Ag Sensor Detects MG Molecules in Complex Environments

**Figure 6:**
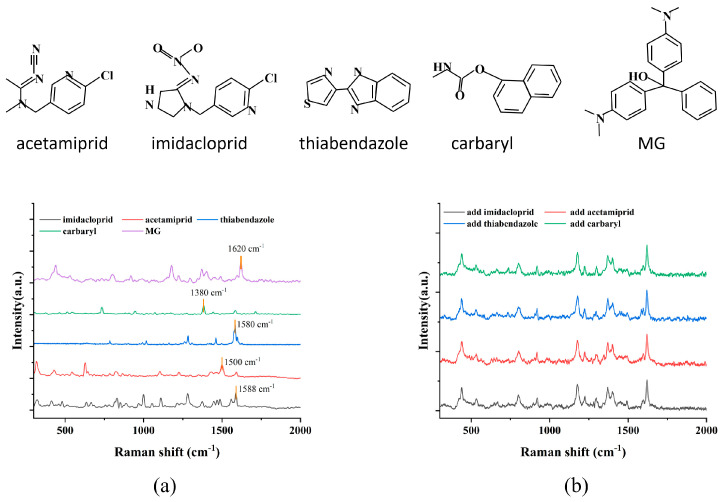
(**a**) Raman spectra of different molecules. (**b**) Spectra of MB detected by G/M/Ag sensor in the mixed solution.

To verify the detection capability of the composite film for MG, four common fungicide molecules—acetamiprid, imidacloprid, thiabendazole, and carbaryl—were selected as interfering substances to conduct an anti-interference experiment in this work. These interfering molecules were widely used in the field of food safety due to their low price and strong sterilization ability. Figure 6a displayed the Raman spectra of acetamiprid [18], imidacloprid [19], thiabendazole [20], and carbaryl [21], with characteristic peaks at 1500, 1588, 1580, and 1380 cm^−1^. Their distinctive peak shapes were significantly different from the characteristic peak of MG at 1620 cm^−1^, which made it easier to identify the MG signal in the mixed solution. In the experiment, 1000 pM MG was sequentially mixed with 5000 pM concentrations of the aforementioned interfering molecules to prepare a series of interference solutions, simulating complex environments. The G/M/Ag sensor was separately immersed in each mixed solution for 40 min, and the SERS tests were performed after it was taken out. As shown in Figure 6b, even when the concentration of interfering substances far exceeded that of MG, the strong characteristic Raman peak of MG could still be clearly detected on the surface of the G/M/Ag sensor, while the signal from the interfering substances was either absent or extremely weak. This result was primarily attributed to MG being a macromolecule with a conjugated structure, which possessed a large scattering interface. The surface structure of g-C_3_N_4_ enabled the preferential capture and enrichment of MG molecules through π–π stacking and electrostatic interactions, which effectively enhanced the Raman signal of MG molecules [22]. In addition, MG exhibited an absorption band near the 532 nm laser wavelength, inducing a resonant SERS effect that further enhanced the SERS signal intensity of MG. This experiment demonstrated that the G/M/Ag sensor exhibited strong anti-interference capabilities and was suitable for detecting trace amounts of magnetic fields in complex environments [23].

### 3.4. G/M/Ag Sensors for Detecting MB in Aquatic Products

**Figure 7:**
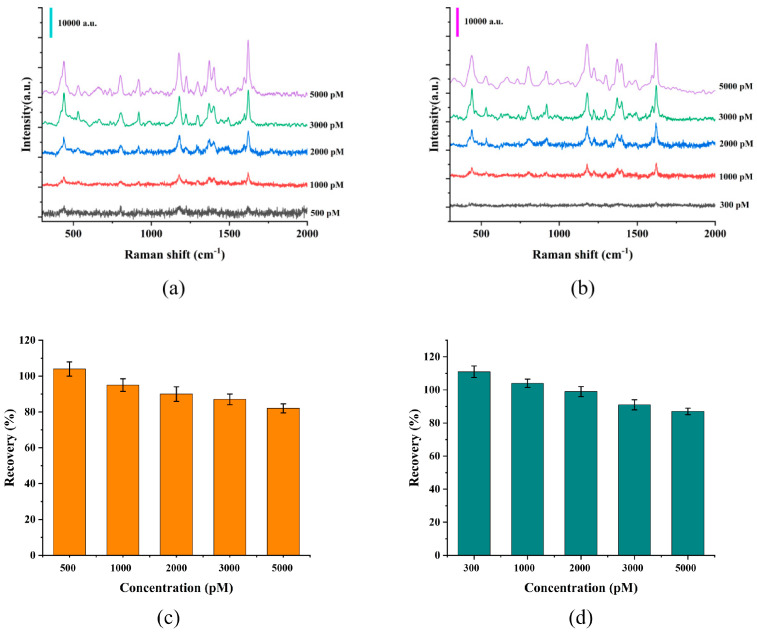
(**a**) The G/M/Ag sensor detected MG in fish skin. (**b**) The G/M/Ag sensor was used to detect MG in river water. (**c**) The recovery rate of MG detected in fish skin. (**d**) The recovery rate of MG detected in river water.

To test the practical application feasibility of the composite film in real samples, fish tissue and river water were selected as real test samples in this work. For river water samples, filter membranes with appropriate pore sizes were used for filtration to remove suspended particles and other impurities that might adhere to the sensor surface and affect the collection of SERS signals, thereby avoiding signal distortion caused by matrix interference. After the sample pretreatment was completed, MG was added to each of them separately to prepare a series of real sample solutions with different concentrations. Subsequently, the sensor was immersed in the prepared sample solutions for 50 min to allow MG molecules to be fully adsorbed on the sensor surface. Figure 7a,b presented the SERS signals collected by the G/M/Ag sensor in fish tissue samples and river water samples with different MG concentrations, respectively. The results showed that the composite film could still effectively identify the characteristic Raman peaks of MG even in river water samples with an MG concentration of only 300 pM. In this work, we calculated the recovery rate by taking the ratio of the concentration of MG detected by SERS to the concentration of MG initially added to the real samples. The results in Figure 7c,d showed the recovery rates of MG detected by the G/M/Ag sensor in fish tissue and river water, indicating that the recovery rates were stable and within the range of 80% to 110%. This fully demonstrated that the composite film possessed excellent detection performance and practical application potential in real environmental systems.

**Figure 8:**
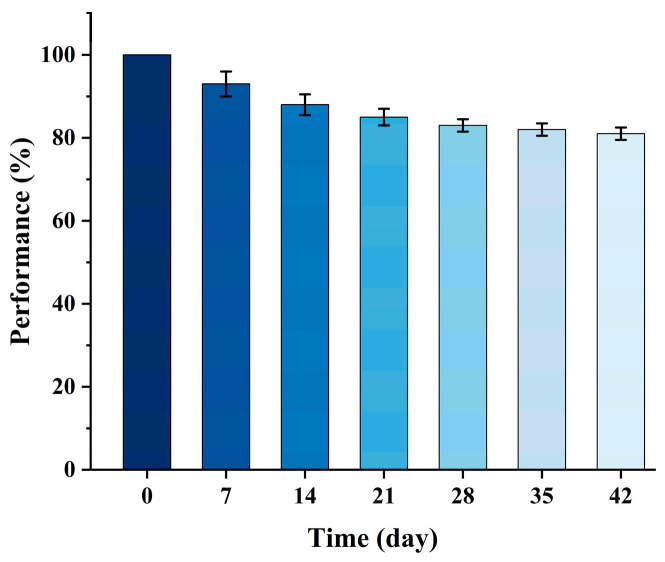
The detection intensity results of 5000 pM MG by the sensor over a period of six weeks.

To investigate the stability performance of the sensor, which is a crucial parameter for its practical application, the sensor was sealed in a vacuum packaging bag and stored under constant temperature conditions (25 ± 1 °C, relative humidity 50 ± 5%) in this work. Subsequently, the sensor was removed every 7 days and immersed in a 5000 pM MG solution for 30 min. After air-drying, SERS signals were collected from the sensor surface. The intensity of the Raman peak at 1620 cm^−1^ was recorded to study the stability of the sensor. Three parallel experiments were carried out for each detection to ensure the reliability of the data, and the resulting signal attenuation trend of the sensor is shown in Figure 8. The analysis demonstrated that the sensor could still maintain 81% of its initial signal intensity after 42 days of sealed storage, indicating its outstanding long-term stability and good anti-interference ability against environmental factors.

To highlight the advantages of the sensor fabricated in this work, Table 1 summarizes the recent relevant studies on MG detection. A comparison of these studies revealed that the G/M/Ag sensor exhibited excellent SERS performance, could achieve highly sensitive detection of MG at low concentrations, and demonstrated great application potential. Combined with its excellent long-term stability mentioned above, the sensor demonstrated great application potential in practical fields such as food safety supervision, environmental monitoring and biological detection.

## 4. Conclusions

In this work, a SERS sensor with a G/M/Ag composite structure was constructed via electrodeposition technology for the highly sensitive detection of MG molecules in aquatic products. This sensor took a 3D nano-network with a high specific surface area as the substrate, which could efficiently enrich target molecules. Meanwhile, the silver nanoparticles uniformly modified on the substrate surface could produce a remarkable LSPR effect, thereby significantly enhancing the Raman signals of MG molecules adsorbed on its surface. The detection performance of the sensor was further improved by systematically optimizing the deposition time of silver nanoparticles and the sample immersion time. After the optimization of sensor preparation and pretreatment conditions, the G/M/Ag SERS sensor exhibited excellent detection performance for aqueous MG solutions. Experimental results demonstrated that the sensor achieved a LOD of 8.8 pM for MG with a linear detection range of 10–5000 pM, and exhibited good accuracy and reliability in the detection of actual aquatic product samples. The G/M/Ag SERS sensor developed in this work provided a sensitive and practical analytical method for the trace detection of malachite green in food products, and possessed considerable potential for practical applications.

## Figures and Tables

**Table 1:** Detection limits of MG by different sensors.

Methods	Sensor	LOD	References
Electrochemistry	TiO_2_/ZnO	0.34 nM	[8]
Electrochemistry	High nitrogen-doped carbon nanosheets	13.2 nM	[9]
Electrochemistry	M_3_(HHTP)_2_	1.34 nM	[10]
SERS	Au@Ag nanorods	1.58 nM	[11]
SERS	MIL-101-MA@Ag	0.09 nM	[12]
SERS	Droplet-based sensor	2.26 nM	[13]
SERS	NPP	0.08 nM	[14]
SERS	G/M/Ag	8.8 pM (0.009 nM)	This work

## Data Availability

The original contributions presented in this study are included in the article. Further inquiries can be directed to the corresponding author.
